# No evidence of *BRCA2 *mutations in chromosome 13q-linked Utah high-risk prostate cancer pedigrees

**DOI:** 10.1186/1756-0500-2-94

**Published:** 2009-05-28

**Authors:** Kristina Allen-Brady, James M Farnham, Nicola J Camp, Eric Karlins, Elaine A Ostrander, Lisa A Cannon-Albright

**Affiliations:** 1 Genetic Epidemiology Group, Department of Biomedical Informatics, University of Utah School of Medicine, 391 Chipeta Way, Suite D, Salt Lake City, UT 84108 USA; 2 Cancer Genetics Branch, National Human Genome Research Institute, National Institutes of Health, Building 50, Room 5351, 50 South Dr, MSC 8000 Bethesda, MD 20892 USA

## Abstract

**Background:**

Germline mutations in the *BRCA2 *gene have been suggested to account for about 5% of familial prostate cancer; mutations have been reported in 2% of early onset (i.e., ≤ 55 years) prostate cancer cases and a segregating founder mutation has been identified in Iceland (999del5). However, the role of *BRCA2 *in high risk prostate cancer pedigrees remains unclear.

**Findings:**

We examined the potential involvement of *BRCA2 *in a set offive high-risk prostate cancer pedigrees in which all prostate cases were no more distantly related than two meioses from another case, and the resulting cluster contained at least four prostate cancer cases. We selected these five pedigrees from a larger dataset of 59 high-risk prostate cancer pedigrees analyzed in a genome-wide linkage screen. Selected pedigrees showed at least nominal linkage evidence to the *BRCA2 *region on chromosome 13q. We mutation screened all coding regions and intron/exon boundaries of the *BRCA2 *gene in the youngest prostate cancer case who carried the linked 13q segregating haplotype, as well as in a distantly related haplotype carrier to confirm any segregation. We observed no known protein truncating *BRCA2 *deleterious mutations. We identified one non-segregating *BRCA2 *variant of uncertain significance, one non-segregating intronic variant not previously reported, and a number of polymorphisms.

**Conclusion:**

In this set of high-risk prostate cancer pedigrees with at least nominal linkage evidence to *BRCA2*, we saw no evidence for segregating *BRCA2 *protein truncating mutations in heritable prostate cancer.

## Findings

Prostate cancer is the most frequently diagnosed cancer, excluding basal and squamous cell skin cancers, and the second leading cause of cancer death in men in the United States. Significant familial clustering of prostate cancer cases has been observed [1,2] suggesting that highly penetrant prostate cancer predisposition genes exist. Although a number of genes have been implicated in prostate cancer (see Review articles [2-4]), most require validation and functional studies to determine their precise role in the etiology of prostate cancer.

One gene that has consistently been associated with prostate cancer is *BRCA2 *(see Review article[5]). Early studies in Iceland identified a *BRCA2 *founder mutation, 999del5, that segregates with prostate cancer particularly in cases diagnosed ≤ 65 years. [6,7] The Breast Cancer Linkage Consortium has reported an increased risk of prostate cancer in families with any known deleterious *BRCA2 *mutation[8] First-degree male relatives of women with breast and/or ovarian cancer and a known *BRCA2 *mutation were found to have an increased risk of prostate cancer (RR = 4.65), and rates were found to be even higher in male relatives less than 65 years of age (RR = 7.33)[8] However, a subsequent study of 22 families with a minimum of three men affected with prostate cancer that also included at least two cases of breast and/or ovarian cancer observed no *BRCA2 *truncating mutations[9] Another common *BRCA2 *founder mutation, 6174delT, has been found to increase prostate cancer risk in some individuals with Ashkenazi Jewish ancestry,[10] but these results have not been confirmed in other studies [11-14]

It has been estimated that germline mutations in *BRCA2 *account for about 5% of prostate cancer in familial clusters[15,16] However, other than the founder mutation in Iceland and possibly another mutation in those with Ashkenazi Jewish ancestry, the role of segregating *BRCA2 *mutations in familial prostate cancer remains unclear. To further assess involvement of *BRCA2 *in familial prostate cancer, we screened for *BRCA2 *variants in high-risk prostate cancer pedigrees that showed at least nominal linkage to the *BRCA2 *region on chromosome 13q.

### Utah Prostate Cancer Resource

The vast majority of studied Utah high-risk prostate cancer families have been identified using the Utah Population Database (UPDB)[17] The UPDB is a computerized database that links cancer information from the Utah Cancer Registry (UCR) to genealogy information for almost four million individuals who are, for the most part, descendants of the nineteenth century pioneers to Utah. The UCR began collecting statewide cancer records in 1966, and in 1973 the UCR became part of the National Cancer Institute's Surveillance, Epidemiology, and End Results (SEER) Program. As a SEER site, all cancers in the state of Utah, except basal and squamous cell skin cancers, are required by law to be reported. Approximately 60% of cancer records in the UCR link to a UPDB genealogy record. The Utah population represented in the UPDB have been shown to be similar to other Caucasian populations of Northern and Western European descent [18] with normal levels of inbreeding[19] On rare occasions, families with an excess of prostate cancer who reside outside of Utah are contributed to the Utah Prostate Cancer High-Risk Pedigree Resource by collaborators.

From the Utah High-Risk Prostate Cancer Pedigree Resource of over 300 sampled pedigrees, we selected a subset of the most informative prostate cancer pedigrees for a genome-wide linkage search. [20] These pedigrees satisfied the following two criteria: all cases were no more distantly related than two meioses from another case and the resulting cluster contained at least four cases. There were no other exclusion criteria for the linkage analysis including age at diagnosis, or other cancers or disease states (e.g., breast cancer) within the family. We did not remove any prostate cancer pedigrees from our High-Risk Prostate Cancer Resource because of prior knowledge of a *BRCA2 *mutation. We note that our high-risk prostate cancer pedigree definition is more strict than the usual definition of a high-risk prostate cancer pedigree[5] We identified 59 pedigrees, from 3 to 6 generations in size, containing 464 cases and an additional 1,261 unaffected relatives. We genotyped 246 of the 464 cases, as well as 640 of the 1,261 unaffected relatives to infer genotypes of deceased relatives or phase for genotyped cases. All enrolled subjects gave informed consent, and this study was approved by the University of Utah Institutional Review Board.

### Linkage analysis

Genotyping was performed by the Center for Inherited Disease Research (CIDR) using a panel of 404 fluorescent microsatellite (STR) markers spaced ~10 cM apart across the genome. The laboratory methods used by CIDR are described in detail at . Mendelian inheritance was verified for all markers, and samples with an inheritance incompatibility were set to missing.

Linkage analysis was performed using MCLINK, [21] a Markov chain Monte-Carlo method that allows for fully informative multilocus linkage analysis on large extended pedigrees, using the Smith *et al*. prostate cancer inheritance model. [22] MCLINK computes the robust multipoint linkage statistic proposed by Göring and Terwilliger, [23] referred to as theta LOD or TLOD[24] The results of this genome-wide linkage scan are reported elsewhere. [20]

We identified 5 pedigrees with at least nominally significant linkage evidence (TLOD score ≥ 0.588) within approximately 10 cM of the *BRCA2 *gene on chromosome 13q12.3 at 24.4–24.6 cM, using the Marshfield map. A TLOD score of 0.588 corresponds to a *p*-value of 0.05, not accounting for multiple testing. Table 1 provides details of the linkage evidence for each pedigree. For two of the five pedigrees (9445402 and 9990205), the highest genome-wide TLOD score was at the chromosome 13q12 region. For the remaining three pedigrees, the chromosome 13q12 peak was the second (9775803), third (9434307) and fourth (9435901) highest of all peaks. For kindred 9775803, the 13q12 peak was preceded by a peak at 2q36-q37. For kindred 9434307, the 13q12 peak was preceded by peaks at 12q24 and 2p25-p24. Finally, for kindred 9435901, the 13q12 peak was preceded by peaks at 18p11-q22, 19q13, and 1p35-p34.

**Table 1:** Characteristics of five high-risk prostate cancer pedigrees showing linkage evidence to chromosome 13q, *BRCA2 *region^1^

Pedigree Identifier^2^	Max TLOD score on chr 13q	Location of max TLOD on chr 13	# prostate cancer cases in pedigree (genotyped)	# prostate cancer cases carrying segregating 13q haplotype^3^	Age at prostate cancer diagnosis (yrs)
9775803	1.423	23.0 cM	9(6)	7	57, 59, 61, 61, 64, 73, 74, 84, 87
9445402	1.191	23.0 cM	5(3)	5	60, 60, 78, 79, 82
9990205	1.050	36.0 cM	10(5)	3	59, 61, 62, 65, 66, 66, 70, 73, 77, 78
9434307	0.976	29.0 cM	12(6)	6, 7^4^	52, 59, 59, 63, 63, 69, 72, 74, 75, 76, 80, 85
9435901	0.953	23.0 cM	6(1)	4	54, 57, 62, 64, 67, 82

^1^Pedigrees defined in Table 1 are based on the pedigree structure used for the linkage analysis.

^2^Pedigree identifier modified to protect confidentiality of pedigree members.

^3^Haplotype carrier status may be inferred based on descendant information rather than directly genotyped.

^4^Two distinct 13q haplotypes segregated within the pedigree.

In each linked pedigree, we selected the youngest prostate cancer case that carried a segregating haplotype of interest as well as the most distantly related individual in the pedigree who also carried the same segregating haplotype for *BRCA2 *mutation screening. One pedigree had two 13q segregating haplotypes; both were screened for mutations. Hence, twelve individuals were selected for *BRCA2 *mutation screening. Ten of the subjects for mutation screening were prostate cancer cases and two of the subjects were unaffected relatives (1 male and 1 female) who carried the 13q segregating haplotype.

### *BRCA2 *mutation screening

All coding regions and intron/exon boundaries of the *BRCA2 *gene were screened for mutations using 47 primer pairs that have been described previously. [25] Shorter exons were amplified in a single fragment and longer exons were amplified as sub fragments. There were five fragments for exon 10, 16 fragments for exon 11, and two fragments for each of the following exons: 14, 18, and 27. PCR reactions were performed in a reaction volume of 10 μl containing 5 ng of genomic DNA, 1 mM dNTPs, 1.5 mM or 2.5 mM MgCl_2_, 1 μM of both the forward and reverse primer, and 0.25 U Amplitaq DNA polymerase (Applied Biosystems, Foster City CA).

PCR products were cleaned using the ExoSAP-IT method (USB Corporation, Cleveland, OH). DNA sequencing reactions were conducted using BigDye^® ^Terminator v3.1 Cycle Sequencing Kits (Applied Biosystems, Foster City CA) and sequence data were obtained on both forward and reverse PCR primers on ABI 3730xl Genetic Analyzer (Applied Biosystems, Foster City CA). Contigs were assembled using PhredPhrap/Consed[26] Polymorphisms were detected using Polyphred [27] and MutationSurveyor [28] by two independent researchers. All variants were unanimously confirmed.

Summary characteristics of the five screened pedigrees are shown in Table 1. In four of the five pedigrees, the linked 13q segregating haplotype was carried by the majority of prostate cancer cases. The age at diagnosis varied from what would be considered an early-onset case (i.e., ≤ 55 years – two prostate cancer cases), to cases with a diagnosis over the age of 80 years (six cases).

Next, for each pedigree we identified the common ancestor hypothesized to carry the 13q segregating haplotype. For all of the descendants of this ancestor, we identified all individuals with diagnosis of a known *BRCA2*-associated cancer (breast cancer, prostate cancer, ovarian cancer, pancreatic cancer, and melanoma) using linked UCR data. These results are shown in Table 2. In each pedigree, the number of prostate cancer cases among descendants of this most distant ancestor significantly exceeded that expected in a population matched by birth year (within five years), sex, and place of birth (Utah versus outside of Utah): p-value range: 0.007–0.000006. For pedigree 9445402, the number of melanoma cases also exceeded the matched population rate (p-value: 0.031). Although four of the five families had at least one known breast cancer case, there were no pedigrees with a significant excess of breast cancer cases. Furthermore, we observed no male breast cancer cases in any pedigree. The Utah Prostate High-Risk Cancer Resource is continually updated from the UPDB with new descendant information and newly diagnosed prostate cancer cases. Differences in the number of prostate cancer cases exist between Tables 1 and 2. Table 1 contains information on the pedigree structure and sampling design used in the original linkage analysis. Table 2 reflects current cancer information for each pedigree.

**Table 2:** Risk of *BRCA2 *associated cancers in descendants of most distant ancestor carrying the 13q haplotype^1^

Pedigree Identifier	# descendants	Cancer type	# of observed cancers	# of expected cancers	p-value
9775803	380	Breast	1	1.41	0.755
		Prostate	9	2.15	**0.0004**

9445402	206	Breast	2	0.79	0.188
		Melanoma	2	0.27	**0.031**
		Prostate	6	0.70	**0.00009**

9990205^2^	NA	NA	NA	NA	NA

9434307	1,863	Breast	5	5.36	0.621
		Melanoma	4	2.25	0.190
		Prostate	22	7.13	**0.000006**
		Ovary	1	0.88	0.585

9435901	204	Breast	1	0.73	0.516
		Melanoma	1	0.30	0.261
		Prostate	5	1.17	**0.007**

^1 ^The following cancers were considered to be *BRCA2 *associated cancers: breast, prostate, ovarian, pancreatic, and melanoma.

^2^Pedigree 9990205 was contributed from a collaborator outside of Utah, and does not link to the UPDB. The number of cancers in descendants from this pedigree cannot be determined.

No known deleterious mutations in *BRCA2 *were observed among the 12 carriers of the chromosome 13q haplotypes. We observed one *BRCA2 *variant of unknown significance as defined by the Breast Cancer Information Core (BIC) [29] database and one variant not listed in the BIC database (see Table 3). The variant of unknown significance (5972C→T) was detected in an unaffected male 1^st ^cousin (last known age to be cancer free: 72 years) but was not carried by the sampled prostate cancer case in the pedigree (diagnosis age: 54 years). The intronic variant not previously reported in the BIC database (IVS14 -62A→G) was observed in a single prostate cancer case, but it was not detected in the other haplotype carrier tested in the pedigree.

**Table 3:** *BRCA2 *variants of unknown significance observed in Utah high-risk prostate cancer pedigrees

Exon/Intron	Nucleotide change	Amino Acid change	Desc. On BIC^1^	No. found in prostate cases /total found /no. with valid results^2^	Pedigree segregation
11	5972C>T	M1915T	UV(7)	0/1/12	--^3^
I14	IVS14-62A>G	Intronic	NA	1/1/12	--

^1^UV = unclassified variant. Value in parentheses represents the number of times there is an entry for the given mutation in the BIC database.

^2^Valid result requires that the sequencing reaction is of sufficient quality as to detect the polymorphism.

^3^This variant was previously reported to be present in prostate cancer cases by others.^21^

We also observed a number of *BRCA2 *polymorphisms that are likely of no biological consequence (see Table 4). Many of these polymorphisms have been reported in other individuals with prostate cancer, and the majority segregated within pedigrees.

**Table 4:** *BRCA2 *polymorphisms observed in Utah high-risk prostate cancer pedigrees

Exon /Intron	Nucleotide change	Amino Acid change	Desc. on BIC^1^	# found in prostate cases /total found /# with valid results^2^	Pedigree segregation	Literature observing same variant in other prostate cancer cases
2	203G>A	5' UTR	P(14)	1/2/12	^--^	^11,19,21,22^
10	1093A>C	N289H	M(9), UV(12)	2/3/12^3^	9434307	^11,19,21,22^
10	1342A>C	N372H	M(9)	5/6/12^3^	9990205	^11,19,21,22^
10	1593A>G	S455S	Syn(7)	2/3/12^3^	9434307	^11,19,21,22^
11	2457T>C	H743H	Syn(4)	2/3/12^3^	9434307	^19,21,22^
11	3199A>G	N991D	M(3)	2/3/12^3^	9434307	^19,21,22^
11	3624A>G	K1132K	Syn(8)	1/2/12	--	^19,21,22^
11	4035T>C	V1269V	Syn(3)	3/3/12	9445402	^19,21,22^
14	7470A>G	S2414S	Syn(10)	1/2/12	--	^11,19,21,22^
15	7772C>T	T2515I	M(70)	1/1/10	--	^16^
I17	IVS17 -14T>C	Intronic	M(15)	5/7/12^3^	9434307 9775803	^21,22^

^1^P = polymorphism, M = missense, UV = unclassified variant, Syn = synonymous. Value in parentheses represents the number of times there is an entry for the given mutation in the BIC database.

^2^Valid result requires that the sequencing reaction is of sufficient quality as to detect the polymorphism.

^3^One of the unaffected carriers of the variant was female who was diagnosed with rectal cancer at age 55 years.

Our results are consistent with a growing pool of evidence that *BRCA2 *plays at most a minor role in causing prostate cancer in high-risk prostate cancer families. As with our results, Sinclair *et al*. [9] using high-risk prostate cancer pedigrees found no *BRCA2 *truncating mutations, but found one previously reported missense mutation and two previously unreported intronic polymorphisms. Gayther *et al*. [16] found two germ-line *BRCA2 *truncating mutations in 38 high-risk, familial prostate cancer pedigree in two individuals diagnosed ≤ 56 years, neither of which was found to segregate. A recent study by Agalliu *et al*. using 266 subjects in high-risk prostate cancer families also reported no disease-associated protein truncating *BRCA2 *mutations[30] Our study is unique in that we focused on pedigrees with strong linkage evidence near the *BRCA2 *gene region on chromosome 13q, and that all pedigrees met a very strict definition of high-risk.

While the five linked pedigrees studied here appear to have minimal involvement of the most likely candidate gene in the region (i.e., *BRCA2*), there is still unexplained linkage evidence on chromosome 13q that cannot be attributable to *BRCA2 *coding mutations. There may be yet unidentified regulatory *BRCA2 *mutations, deletions, and/or rearrangements that were not screened as part of this study. Alternatively, the nominal linkage evidence in these prostate cancer pedigrees may represent other prostate cancer predisposition genes located near *BRCA2 *including *HMG1*, *LGR8*, *CCNA1*, *TRPC4*, and *FOXO1*. Finally, the results may be spurious as only a limited number of pedigrees (i.e., five of the original 59 pedigrees) were studied, and segregation analysis with chromosome 13q may not be the most reliable way of identifying *BRCA2 *mutation families.

## Conclusion

This study adds further evidence that *BRCA2 *has a limited role in heritable prostate cancer. We observed no evidence for *BRCA2 *coding mutations segregating within our Utah high-risk prostate cancer pedigrees with prior linkage evidence to chromosome 13q.

## List of Abbreviations

RR: relative risk; OCCR: ovarian-cancer cluster region; UPDB: Utah Population Database; UCR: Utah Cancer Registry; SEER: National Cancer Institute's Surveillance, Epidemiology, and End Results Program; CIDR: Center for Inherited Disease Research; STR: short tandem repeat/microsatellite marker; TLOD: theta LOD; BIC: Breast Cancer Information Core

## Competing interests

LCA: Receives royalty monies from Myriad Genetics from *BRCA2 *testing because of her work to identify the relationship between *BRCA2 *and an increased risk of breast cancer.

All other authors declare that they have no competing interests.

## Authors' contributions

KAB: participated in the design and coordination of the study, statistical analysis and drafted the manuscript; JMF: participated in the design and coordination of the study; NJC: participated in the study design; EK: performed the genetic sequencing; EAO: participated in the study design and helped to draft the manuscript; LACA: conceived of the study, participated in the design and coordination and helped draft the manuscript. All authors read and approved the final manuscript.
